# Institutional Notes and News

**Published:** 1910-07-16

**Authors:** 


### Institutional Notes and News.

In our issue of June 25 an account appeared of the opening and public inspection of the new buildings at the East Suffolk and Ipswich Hospital. Since then the annual meeting has been held, and, in view of the structural alterations which have taken place, the general condition of the hospital and its work is worth notice. Patients in both departments have increased in number, and more beds have been filled than ever before in the three-quarters of a century of the hospital's existence. An analysis of the sources of income shows that legacies during the past year have dwindled almost to vanishing-point, that subscriptions have maintained their position with a struggle, but that the contributions from the Employé's Fund have more than replaced the sum lost by the smallness of the legacies. The figures give an added point to this: Legacies fell from £236 to £29, and the Employé's Fund rose from £983 to £1,367. It should be added that another bed has been endowed by means of £1,000 left by the late Mrs. Burley for that purpose. The death of the late president, Mr. Felix T. Cobbold, has removed a man who held that office for twelve years, and who was ever generous in his support of the institution. His memory is to be honoured by christening one of the men's wards after him. The name of his successor, Dr. J. H. Bartlett, just re-elected for the year, has been mentioned already in our "Hospital World."

**East Suffolk and Ipswich Hospital.**

The following is taken from the current annual report of the Wolverhampton and Midland Counties' Eye Infirmary: "A visit of inspection was paid to the Eye Infirmary by Sir Henry Burdett, K.C.B., K.C.V.O., a well-known hospital authority, who reported that he 'found the hospital in good order throughout.' In the report of his visit in The Hospital he suggested a slight alteration, which has been carried out." The "slight alteration" suggested in the report was the removal from the operation theatre of two beds, one of which was used habitually, in the words of our report, "so that other children may be operated upon in the theatre whilst the nurse and matron attend to children in the beds as well as to the child on the table." The report, which is to be found in our issue of October 30, 1909, page 132, pointed out that this practice of retaining "patients who have been operated upon in a theatre whilst an operation on another patient is proceeding" was indefensible. As all this has been changed it is perhaps not worth while to argue whether it was a "slight alteration" or not, in any case, we congratulate the authorities on remedying a defect which, sooner or later, must have brought discredit to the hospital.

**Wolverhampton Eye Infirmary.**

The Committee of the South Wimbledon Cottage Hospital has decided to build a new hospital, and after much difficulty has secured a site at The Rush, Merton, an amalgamation with the North Wimbledon Hospital Committee for a large hospital on a central site having long been discussed in vain. Last week the plans sent in were assessed by Mr. C. W. Pike, the nominee of the president of the R.I.B.A., who adjudged the best to be that of Mr. Francis Hatch, of 71 Queen's Road, Wimbledon. The estimated cost for a hospital of twenty-four beds was given at £6,118. In reply to the secretary of the committee, Mr. H. M. Ellis, S.C.C., surveyor, Mr. Hatch said he was engaged at the War Office, and a discussion took place as to whether he would be able to devote sufficient time to the hospital outside his service duties. Mr. Hatch told the committee he was entitled to thirty-six days' leave, and there would be no difficulty. The Chairman stated that £1,350 was paid for the site, and the funds now available were £2,800, but an anonymous friend offered a loan of £1,000 without interest, and £600 was promised as a memorial gift in memory of the late Mr. Carver, an old friend of the hospital.

**New Hospital, Wimbledon.**

The new operation-theatre at the Whitworth Hospital, Darley Dale, and the nurses' home have now been opened. Dr. W. Moxon, J.P., the chairman of the institution, gave an interesting account of the origin of the hospital. He came to Matlock, he said, in 1878, and for ten years the district possessed no means of treating the sick. He made every effort to secure the building of a cottage hospital, but, in spite of a great deal of talking, the money for the purpose could not be raised. One day, several years later, Dr. Moxon attended one of Lady Whitworth's servants, and his account of the man's condition and needs inspired Lady Whitworth to build a cottage hospital. She consulted Dr. Moxon as to its site, which was the one on which the present hospital stands to-day. The hospital was opened in 1889, and was, in fact, by whatever name it might be called, said Dr. Moxon, a memorial to her ladyship. The present additions mean that sixteen beds have to be maintained, and it is hoped that the public spirit of the district will remember the difficulties that have been conquered in the hospital's past, and will provide the money for its efficient upkeep. Mr. C. E. Dawson is the architect.

**Whitworth Hospital, Darley Dale.**

The Lord Mayor of Bristol, Mr. C. A. Hayes, and the Lady Mayoress visited the Ham Green Infectious Hospital last week. The visit has real interest because it marks the progress and development of this municipal hospital, and the results which followed from the Corporation's acceptance of the site. It was opened in 1899, and its history was given by Dr. Colston White in a short speech. It was purchased, he said, by the late Sir George Edwards in 1893, but knowing that the city wanted a place for an infectious hospital, he allowed them to have it at the same price he had paid—namely, £8,600. The site contains some ninety-nine acres, and thirty-eight of these are used for hospital purposes. In 1905 the committee built an additional wing: a two-storied building, which provides fifty-eight beds, bringing the total up to 135. In times of stress they had had as many as 200 cases, and at the present time 150, that being the average for the last two or three years. Ten years ago medical men had considerable difficulty to get patients to come there, but the only difficulty now was to prevent overcrowding. They had an excellent matron, and the best medical man they could get as resident medical officer under the supervision of Dr. Davies. They had also capable trained nurses, and in so doing they were carrying out their work as a health committee and protecting their fellow-citizens against the spread of infectious disease. The splendid gardens are famous for grapes and tomatoes, and supply the needs of the patients; the remainder not required being sold in Clifton. The Lord Mayor congratulated the city on seizing the opportunity when it occurred of purchasing the site.

**Ham Green Infectious Hospital.**

